# Extensor indicis transfer versus palmaris longus transplantation in reconstruction of extensor pollicis longus tendon: a protocol for a systematic review

**DOI:** 10.1186/s13643-020-01409-3

**Published:** 2020-06-23

**Authors:** Abdulwares Meiwandi, Sarantos Kaptanis, Marios Papadakis

**Affiliations:** 1 Department of Plastic, Reconstructive, Aesthetic and Hand Surgery, Helios University Hospital Wuppertal, Witten-Herdecke University, Wuppertal, Germany; 2 Queen Mary University of London, London, UK; 3 Division of Surgery II, Witten-Herdecke University, Wuppertal, Germany

## Abstract

**Abstract:**

This review will provide an overview of published data comparing transposition of the extensor indicis pollicis (EIP) tendon and palmaris longus (PL) tendon grafting in thumb extension reconstruction following loss of the extensor pollicis longus (EPL) tendon function. We will consider all studies comparing EIP and PL utilized to reconstruct thumb extension after injury/rupture of the EPL tendon. Only studies published in the English and German literature will be included. A systematic literature research will be performed across relevant health databases including the Cochrane Library, MEDLINE, and Google Scholar using the following keywords: ((extensor pollicis longus) OR EPL) AND ((extensor indicis) OR EIP OR (tendon transposition)) AND ((palmaris longus) OR (free tendon graft) OR (tendon transplantation)). Central tendencies will be reported in terms of means and standard deviations where necessary. If not reported, the standard deviation will be calculated from the standard error of the mean. Risk ratios will be calculated where possible. All calculations will be performed with a 95% confidence interval. Statistical significance will be set at *P* < 0.05. Adjusted effect estimates will be analyzed in preference to the unadjusted estimates, using inverse-variance weighted average. Pooled estimates will only be presented after consideration of both clinical and methodological heterogeneity of included studies. The review will be reported using the Preferred Reporting Items for Systematic Reviews and Meta-Analysis (PRISMA) statement.

**Systematic review registration:**

The protocol was registered on PROSPERO CRD42019135735: https://www.crd.york.ac.uk/prospero/display_record.php?RecordID=135735

## Introduction

### Rationale

The extensor pollicis longus (EPL) muscle is the most important extensor of the IP and MCP joint of the thumb. It originates from the dorsal distal third of the ulna and membrane interossea. The tendon crosses to the radius and enters the third compartment of the extensor retinaculum. It finally inserts at the dorsal base of the distal phalanx. It is the only muscle that allows retropulsion/hyperextension of the thumb [1]. When injured, significant loss of range of motion is noted that cannot be compensated otherwise [2]. The most common cause of rupture of the EPL tendon is sharp trauma. Other reasons include subcutaneous rupture due to tenosynovitis, impaired blood supply, or repetitive mechanical irritation/stress, e.g., after radius/scaphoid fractures or after osteosynthesis at the distal radius [3–6]. Diagnosis is often made through clinical examination or surgical exploration. In unclear cases, sonography or MRI can help verify the diagnosis.

Treatment depends on the cause of injury and on the time of presentation or time of correct diagnosis after rupture. Immediate treatment of sharp injuries allows primary reconstruction of the tendon by primary tendon suture [7]. In cases of delayed presentation/diagnosis or in cases of degenerative ruptures, the tendon stumps are not suitable for primary suture as a considerable defect is often present because of tendon retraction. In these cases, secondary reconstructive procedures are indicated. The most commonly described technique in the literature is the transposition of the extensor indicis proprius (EIP) tendon to the thumb [8–12]. This procedure sacrifices one of the extensor tendons of the index finger and reattaches it to the distal stump of the extensor pollicis longus tendon. The functional deficit that results from this procedure is negligible in patients without high demands. Advantages are shorter operating time, relatively easier technique, and lower rates of postoperative complications such as avascular necrosis and tendon adhesions [8–12]. The other preferred method is reconstruction through an intercalated free tendon graft, e.g., a free palmaris longus (PL) tendon graft [2, 13–17]. The palmaris longus tendon is almost always used as a source of a free tendon graft in the hand as it produces no functional deficit when removed since it only primarily extends the lower part of the palmar aponeurosis [2, 13–17]. Disadvantages of the free tendon graft are that it requires an extended preparation and at least two tendon weave sutures of the proximal and distal stump of the extensor pollicis longus tendon [2, 13–17]. This is believed to result in more postoperative complications such as tendon adhesions [2, 13–17]. A free tendon graft always carries the risk of avascular necrosis. The advantages of this method are the preservation of uninjured motor units and the lack of any retraining. This procedure is often the first choice in patients with high demands of functional restoration and preservation of the index finger [2, 13–17].

Prognosis is overall very good after reconstruction as in nearly all cases [primarily and secondarily] function can be restored [2, 7, 8, 10, 17–19]. Functional outcomes are often assessed using the Geldmacher scheme or DASH score [9, 20, 21].

In our extensive literature search, we could not find any systematic review or meta-analysis that compared the two most commonly used secondary reconstruction methods of the extensor pollicis longus muscle.

This systematic review will, therefore, compare the outcomes of patients without rheumatoid arthritis that were either treated with a transposition of the extensor indicis proprius tendon to those similar patients treated with a free intercalated palmaris longus tendon graft.

### Objectives

This review will provide an overview of published data comparing transposition of the EIP tendon and PL tendon grafting in thumb extension reconstruction following loss of the extensor pollicis longus tendon function.

## Methods

The systematic review will be conducted in line with the PRISMA guidelines [22]. The protocol will be reported in line with the PRISMA-P 2015 checklist [23].

### Eligibility criteria

#### Study types

We will consider all existing studies comparing EIP and PL utilized to reconstruct thumb extension after injury/rupture of the extensor pollicis longus tendon. Studies that examine only one type of intervention without a comparison group will not be considered. If no randomized controlled trials (RCTs) exist on this topic, this systematic review will be based on observational studies. Only studies published in the English and German literature will be included. Patients with rheumatoid arthritis will be excluded from the study, due to the altered anatomy and physiology. As both methods are classic and good established alternatives in EPL reconstruction, the search will not be limited by date.

#### Participants

All patients included in the above eligible studies will be analyzed without restriction.

#### Interventions

EIP and PL tendon graft represent the interventions to be compared.

- EIP refers to tendon transposition utilizing the extensor indicis proprius tendon, which primarily extends the index finger.
- PL tendon graft uses the palmaris longus tendon, which primarily extends the lower part of the palmar aponeurosis. It serves almost always as the first choice for tendon grafting, because of its length and diameter and the fact that it can be used without producing any functional deformity.

### Information sources

A systematic literature research will be performed across relevant health databases including the Cochrane Central Register of Controlled Trials (CENTRAL), Pubmed, and EMBASE.

### Search strategy

The search strategy will include a combination of the following keywords: extensor pollicis longus AND (extensor indicis or tendon transposition) AND (palmaris longus OR free tendon graft OR tendon transplantation). The reference lists of eligible articles will be hand-searched. Additionally, we will search the ClinicalTrials.gov and the European Union Clinical Trials Register for any pending trials.

### Study records

#### Selection process

Study eligibility will be screened and assessed according to inclusion criteria by two independent investigators AM and MP. A detailed full-paper assessment will be performed for each study deemed eligible for inclusion. This will be done independently and unblinded by AM and MP. Any disagreements on eligibility will be discussed, and if required, the third investigator (SK) will be consulted. If necessary, the corresponding authors of eligible articles will be contacted with written requests for additional information.

#### Data collection process

A customized data extraction sheet will be used for data extraction. AM and MP will independently extract data from eligible studies. The following information will be extracted:

- Study design
- Publication language, year, and country of origin
- Size of the study population
- Demographical features: age, sex
- Injury-related data (injury causes, interval between injury and reconstruction)
- Type of intervention: EIP and PL
- Duration and type of follow-up
- Functional outcomes (i.e., DASH score and Geldmacher Scheme)
- Complication rate (rerupture, post-surgical site infections)
- Re-intervention: type and reasons of re-intervention
- The corresponding authors of all eligible articles will be contacted with written requests for additional information, if necessary

### Outcomes and prioritization

#### Primary outcomes

We specify the functional outcome based on the Geldmacher scheme as the primary outcome of interest.

#### Secondary outcomes

Secondary outcomes include re-interventions following reconstruction. Additional secondary outcomes are re-ruptures within 1 year and surgical site infections that are defined by the authors within 30 days post-op as well as the functional outcome based on the DASH score.

### Risk of bias in individual studies

AM and MP will assess risk of bias of all the studies included. For randomized trials, if any, we will assess RoB using the Cochrane RoB tool. The ROBINS-I (“Risk Of Bias In Non-randomised Studies - of Interventions”) tool [24] will be used to assess the quality of non-randomized studies.

### Data synthesis

We will use a random-effects model to synthesize the results as between-study variation due to patient, surgeon, and hospital variability is expected. We will use the R packages meta [25] and metafor [26] to perform statistical analyses. Central tendencies will be reported in terms of means and standard deviations where necessary. If not reported, the standard deviation will be calculated from the standard error of the mean. Risk ratios will be calculated where possible. All calculations will be performed with a 95% confidence interval. Statistical significance will be defined as *P* < 0.05. Adjusted effect estimates will be analyzed in preference to the unadjusted estimates, using inverse-variance weighted average. Pooled estimates will only be presented after consideration of both clinical and methodological heterogeneity of included studies. The unit of study will be patients with loss of EPL function who undergone either EIP transposition or PL free tendon graft.

#### Assessment of heterogeneity

The chi-square test with a *P* value of 0.05 will be used to explore heterogeneity, while the quantity of heterogeneity will be assessed using *I*^2^ statistics. *I*^2^ will be interpreted according to the guidance for Cochrane reviews. It is expected that a random effects model will be used to build up the meta-analysis.

#### Subgroup analyses

Subgroup analyses will be performed for the primary outcomes stratified by gender, age, and injury cause if possible. For example, retired patients are more likely to have a degenerative rupture, whereas rupture secondary to osteosynthesis for distal radial fracture is more likely to occur to active workers, as non-operative treatment is preferred in elderly patients. Similarly, subgroup analyses stratified by injury cause distinguish between degenerative ruptures, osteosynthesis-related ruptures, and delayed presentations.

#### Sensitivity analysis

Sensitivity analysis will be performed to quantify the effect of unmeasured confounding on the results of our meta-analysis (e.g., influence of physiotherapy scheme and duration), as implemented in the R package, EValue [26].

### Meta-bias

We plan to use visual inspection of the funnel plots to assess reporting bias if sufficient studies are available. Possible sources of any funnel plot asymmetry will be explored since there may be publication bias but no asymmetry.

### Confidence in cumulative evidence

The strength of evidence will be assessed using the Grading of Recommendations Assessment, Development and Evaluation (GRADE) approach [27]. Two review authors independently will rank the quality of the evidence as “high,” “medium,” “low,” or “very low.”

## Discussion

Secondary reconstruction of the EPL tendon after degenerative rupture or delayed treatment is a common problem for the hand surgeon, and the correct choice of treatment can sometimes be challenging to decide. While EIP is generally offered to the general undemanding population, the right treatment of more demanding patients, e.g., musicians, sometimes requires more extensive reconstructive measures. The use of a free PL tendon graft seems to be the primary choice in these patients. However, there is no level I evidence to support that recommendation.

This systematic review will compare the outcomes of patients that were either treated with a transposition of the EIP tendon to those similar patients treated with a free intercalated PL tendon graft. The aim is to produce clinical evidence to help decide when to use which method.

## Abbreviations

EIP: Extensor indicis proprius
EPL: Extensor pollicis longus
PL: Palmaris longus
